# HPV11 E6 mutation by overexpression of APOBEC3A and effects of interferon-ω on APOBEC3s and HPV11 E6 expression in HPV11.HaCaT cells

**DOI:** 10.1186/s12985-017-0878-2

**Published:** 2017-11-03

**Authors:** Yongfang Wang, Xinyu Li, Shasha Song, Yang Sun, Jiafen Zhang, Changming Yu, Wei Chen

**Affiliations:** 1 Department of Pharmacology, Institute of Dermatology, Chinese Academy of Medical Sciences and Peking Union Medical College, 12 Jiang Wang Miao Street, Nanjing, 210042 China; 2 Laboratory of Vaccine and Antibody Engineering, Beijing Institute of Biotechnology, Beijing, 100071 China

**Keywords:** Condylomata acuminata, Human papillomavirus 11, HaCaT keratinocytes, Apolipoprotein B mRNA-editing enzyme catalytic polypeptide-like 3, Early gene 6, Interferon-ω

## Abstract

**Background:**

Condyloma acuminatum, infected by low-risk human papillomaviruses (e.g., HPV6 and HPV11), is one of the most widespread sexually transmitted diseases. Apolipoprotein B mRNA-editing enzyme catalytic polypeptide-like 3 proteins (APOBEC3s, A3s) are cellular cytidine deaminases acting as antiviral factors through hypermutation of viral genome. However, it remains unknown whether A3s results in HPV11 gene mutations and interferon-ω (IFN-ω) exhibits antiviral activities through the A3s system. Here we investigated whether enhanced APOBEC3A (A3A) resulted in the E6 gene mutations and explore the effects of recombinant human interferon-ω (rhIFN-ω) on A3s/E6 expression in HaCaT keratinocytes containing the genome of HPV 11 (HPV11.HaCaT cells).

**Methods:**

A3A-overexpressed HPV11.HaCaT (A3A-HPV11.HaCaT) cells were established by lentiviral infection and verified by immunofluorescence and western-blotting. Cell cycle, E6 gene mutations, APOBEC3s/E6 gene expression and subcellular localization were detected by FACS, 3D-PCR and sequencing, qRT-PCR and immunofluorescence respectively.

**Results:**

The results suggested that A3A-HPV11.HaCaT cells were successfully established. Enhanced A3A induced S-phase arrest, G > A/C > T mutations and obvious reduction of E6 mRNA expression. A3A/A3B mRNA expression was up-regulated at 6 h and 12 h and obvious A3A staining existed throughout HPV11.HaCaT cells after rhIFN-ω treatment. RhIFN-ω could also inhibit mRNA expression of HPV11 E6 significantly.

**Conclusions:**

Enhanced A3A repressed HPV11 E6 expression through gene hypermutation, and rhIFN-ω might be an effective agent against HPV11 infection by up-regulation of A3A.

## Background

Human papillomaviruses (HPVs) are a group of small, double-stranded DNA viruses and display strict tissue specificity [1]. They only infect mucosal or cutaneous epidermal tissues of humans and cause a wide range of apparent epithelial lesions [2]. According to oncogenic potential, HPVs can be classified as the high-risk (HR) and low-risk (LR) genotypes. HR HPVs (e.g., HPV16, HPV18) are commonly associated with cervical cancers, while LR HPVs (e.g., HPV6 and HPV11) mainly induce benign lesions, such as condylomata acuminate (CA) [3]. CA has become one of the most widespread sexually transmitted diseases and present a serious threat to social public health because of the increasing incidence and the high recurrence rate throughout the world [4]. It means that HPVs had evolved different ways to avoid detection and clearance by both the innate and adaptive immune system, leading to recurrent and protracted illness [5, 6]. LR HPVs are self-limiting and will usually be cleared by the host immune system in most infections. However, among susceptible populations, such infections tend to become persistent, a likely prerequisite for malignant progression [7]. Thus, the interaction of viral and host immune status especially at the specific sites of infection may play important roles in disease susceptibility and progression of CA.

When LR HPVs invade epithelium through skin wounds, basal keratinocytes are the primary targets of HPVs infection. Recent study reinforces the importance of the keratinocytes as immune sentinels in producing innate immune mediators, acting as non-professional antigen-presentaing cells and instigators of inflammation [8].

The family of Apolipoprotein B mRNA-editing enzyme catalytic polypeptide-like 3 proteins (APOBEC3s, A3s) plays an important roles in innate immune system [9]. The family comprises seven members: A, B, C, DE, F, G and H [9]. The A3s system is widely expressed in different tissues and cell types, especially dendritic cells, macrophages, CD4^+^ T cells and keratinocytes [10–12]. These members can edit single-stranded DNA (ssDNA) and/or RNA substrates of different viruses by converting cytidines to uridines (C to U) or deoxycytidines to deoxyuridines (dC to dU) [9]. They act as potent innate antiviral factors against exogenous viruses such as HIV, HBV and HPV [12, 13]. The family may also induce mutation clusters in different types of cancer, for example, cervical, bladder and breast cancers [14]. Recently, APOBEC3A (A3A) has been reported to be strongly correlated with the integration of HPV DNA which is a crucial step in HPV-induced carcinogenesis [15]. Hence, cellular A3s act as antiviral restriction factors, but may also result in an increase in chromosomal instability.

Among this A3s family, A3A, A3B, A3C and A3H are expressed and localized in cytoplasm and/or nucleus of human cutaneous keratinocytes [12, 13, 16]. The antiviral effects of A3s proteins have been reported with HPVs. Hyperedited HPV1a and HPV16 genomes were found in the specimens from HPV1a plantar warts and HPV16 precancerous cervical biopsies [12]. Zhe Wang et al. also reported that endogenous A3A and A3G induced by IFN-β resulted in early gene 2 (E2) hypermutation of HPV16 in W12 cells [16]. Then the group further demonstrated that hypermutation in the E2 gene of HPV16 existed in cervical intraepithelial neoplasia, which might be caused by A3s proteins [17]. In invasive cervical cancer, the viral DNA of HR HPV is frequently integrated into the cellular host DNA, which often resulted in loss of the viral E2 gene, high-level expression and stability of transcripts encoding the E6/E7 oncoproteins [18]. These oncoproteins are the primary viral factors to initiate and promote tumorigenesis by manipulating cell cycle regulators and inducing DNA damage and chromosomal aberrations [19]. In contrast to HR HPVs, the E6 proteins from LR HPVs types don’t have transforming activity and/ or immortalization capacity as that of HR HPV types does [20, 21]. This E6 protein plays a clear role in the initiation of viral DNA replication and are responsible for the pathogenicity of LR HPV types [20, 21]. Surprisingly, genetic variations of HPV11 E6 were also observed in HPV11 DNA isolated from the clinical specimens of recurrent respiratory papillomatosis, genital warts, anal cancer and cervical neoplasia cells [22], but the mechanism involved in these variants has not yet been well defined. Our previous study showed that HPV11 could up-regulate A3s expression (especially A3A) in HaCaT keratinocytes containing the genome of HPV 11 (HPV11.HaCaT cells) [23]. Thus, we speculated that HPV11 E6 gene might be vulnerable to editing by cytidine deaminases of the A3s family in cutaneous keratinocytes.

The family of type I interferons (IFNs) consists of IFN-β, IFN-ε, IFN-κ, IFN-ω and 12 subtypes of IFN-α. They are produced by virus-infected cells and have been reported to exhibit anti-proliferative, immunomodulatory and antiviral activities [24]. In our previous work, recombinant human interferon-α (rhIFN-α) showed significant inhibition of HPV11 E6/E7 mRNA expression [25] and induced higher A3A in HPV11.HaCaT cells [23]. Human IFN-ω, like other IFNs is secreted from cells in response to viral infection and reported as an antiviral agent for treatment of several viruses such as HCV [26], influenza viruses [27], HBV [28]. IFN-ω has a 60-65% homology to human IFN-α1 and has distinguishable biological properties in type I IFNs [29]. For example, glycosylated IFN-ω has an unique antagonism on the cytotoxic effects of ribavirin that was not observed with IFN-α [29]. This interferon appeared more efficient than IFN-α2 against influenza viruses [27] and showed similar antiviral potential as IFN-α2 on HBV replication in human hepatoma cells [28]. In our laboratory, we also explored the potential antiviral effects of recombinant human interferon-ω (rhIFN-ω), which has shown obvious effects of anti-herpes simplex virus (data not shown). Thus, IFN-ω, used by itself or in combination with other antiviral therapies, might provide a useful alternative for patients who fail to respond to IFN-α or as an additional treatment option [26]. However, studies of type I IFNs have mainly focused on the subtypes of IFN-α and IFN-β, knowledge of IFN-ω is limited. To date, only a few studies have been made to elucidate the possible mechanisms of IFN-ω-dependent antiviral activities. It remains unknown whether IFN-ω exhibits antiviral activities through the A3s system.

In this context, we investigated whether enhanced A3A could induce hypermutation in HPV11 E6 gene by using differential DNA denaturation polymerase chain reaction (3D-PCR) that can selectively amplify A3s-edited viral DNA [12], and then explored the effects of exogenous rhIFN-ω on the expression of A3s and E6 in HPV11.HaCaT cells.

## Methods

### Cell culture

HPV11.HaCaT cells were established and cultured as described previously [30]. HaCaT cells were routinely cultured and passaged by trypsinization using conventional methods.

### Overexpression of A3A and lentiviral infection

Because the established HPV11.HaCaT cells stably maintained HPV11 episomes in passage 15 [30], we chose HPV11.HaCaT (passage 3, P3) in follow-up experiments for continuous research. A3A overexpression in HPV11.HaCaT cells was established using a lentivector-based ORF system according to the manufacture’s instructions. The lentiviral vetor overexpressing human A3A was named EX-I1284-Lv205 and from GeneCopoeia Inc.(Rockville, MD, USA). The high lentiviral particles titers were prepared with Lenti-PacTM HIV packaging mix (GeneCopoeia Inc., Rockville, MD, USA) in HEK 293 T cells. HPV11.HaCaT (P3) were seeded in twenty four-well plates at a density of 5 × 10^4^ per well for 24 h prior to infection. Lentiviral infection was operated by using lentiviral suspension [5.02 × 10^8^ IU/mL, multiplicity of infection (MOI) = 140] according to a standardized protocol. After allowing cells to incubate for 72 h, HPV11.HaCaT cells were passaged twice per week with growth medium (10% FBS) containing 4 μg/mL puromycin to select cells expressing the transduced vector until generating stable-expressed cells which we called A3A-HPV11.HaCaT.

### Immunofluorescence

HaCaT, HPV11.HaCaT and A3A-HPV11.HaCaT were cultured overnight on some coverslips, which were in 3 cm petri dishes. HPV11.HaCaT cells were treated for 6 h with indicated concentrations of rhIFN-ω (provided by Laboratory of Vaccine and Antibody Engineering, Beijing Institute of Biotechnology, Beijing, China). Immunofluorescence was used to analyze protein expression, as previously described [23, 25]. The antibodies used in this study were: rabbit anti- PHO1 (A3A, 1:250 dilution in blocking buffer; Abcam, Cambridge, USA), goat anti-rabbit IgG-conjugated with Alex Fluor 488 (1:400; Beyotime, Shanghai, China). Cells were stained with DAPI solution (3 μg/mL in PBS; Beyotime, Shanghai, China) to visualize nuclei. Finally, fluorescent staining was visualized with laser scanning confocal microscope (Olympus, Tokyo, Japan) with 40× magnification. In the fluorescence image, cytoplasm displayed as green fluorescence and the nucleus displayed as blue.

### Western blotting

Lysates of HaCaT, HPV11.HaCaT and A3A-HPV11.HaCaT cells were prepared by conventional methods [25]. Potein concentrations were measured by BCA protein assay kit. Western blotting was used to analyze protein expression, as previously described [25]. The antibodies used in this study were: rabbit polyclonal GAPDH (1:300; Goodhere, Hangzhou, China), rabbit anti- PHO1 (A3A, 1:750; Abcam, Cambridge, USA), horseradish peroxidase (HRP)-conjugated goat anti-rabbit IgG (1:5000; SAB, Maryland, USA). SuperSignal West Pico Chenilunminiscence Substrate (Pierce Biotechnology, Rockford, IL, USA) was used for detection antibodies and menbranes were scanned for the quantification using Bio-Rad Quantity One software.

### FACS analysis

HaCaT, HPV11.HaCaT and A3A-HPV11.HaCaT cells were plated at a density of 1 × 10^6^ cells/well in six-well plates. After 24 h, they were digested with 0.01 M PBS-0.5% trypsin (1:1). The collected cells were washed with PBS, and then fixed with 70% ice cold ethonalto. The samples, stored at −20 °C, were tested by fluorescene-activated cell sorting (FACS).

### 3D-PCR Amplication, clone, and sequencing

HPV11.HaCaT (P3 and P8) and A3A-HPV11.HaCaT cells were seeded in six-well plates (1 × 10^6^ cells/well). After 24 h, total cellular RNA was extracted from the cell samples using TRIZOL reagent (Invitrogen, Carlsbad, CA, USA) and the concentration and purity of RNA were determined by using NanoDrop (Thermo, USA). The cDNA was synthesized using Reverse Transcription System (Promega, Madison, WI, USA). Circularized HPV11 DNA was prepared [30] and stored in −20 °C. Hypermutated genomes were identified by 3D-PCR in a two-round procedure. RT-PCR was performed in the first round using 2× SYBR Green PCR Master Mix (ABI, Texas, USA) and specific primers of HPV11 E6 (Sangon Bio., Shanghai, China), as previously described [25]. The primers for 3D-PCR are described in Table 1. Differential amplification occurred in the second round, using the 5 μL of the first-round products as input in a final volume of 50 μL. Cycling conditions were 86 °C for 10 min, followed by 35 cycles (86 °C for 45 s, 50 °C for 30 s, and 65 °C for 38 s), and finally for 10 min at 65 °C. Second-round PCR yielded a 453-bp fragment. PCR products were purified from agarose gels and ligated in the TOPO TA cloning vector. After transformation of XL1 Blue cells, three clones from the samples of circularized HPV11 DNA, HPV11.HaCaT (P3 and P8) and A3A-HPV11.HaCaT cells were picked and sequenced. Sequencing analysis was performed by Genscript Corporation (Nanjing, China).

**Table 1:** Oligonucleotide primers used in PCR assays

Gene target	Primer	Sequence(5′-3′)
APOBEC3A	Forward:	5′-GAA GGC AGA GAC CTG GGT TG-3′
	Reverse:	5′-CAT ACT GCT TTG CTG GCG TC-3’
APOBEC3B	Forward:	5′-CCT ACT TGT GCT ATG AGG TGG AG-3′
	Reverse:	5′-TGC AAA GAA GGA ACC AGG TC-3’
APOBEC3C	Forward:	5′-AGC GCT TCA GAA AAG AGT GG-3’
	Reverse:	5′-AAG TTT CGT TCC GAT CGT TG-3’
APOBEC3H	Forward:	5′-GCT TCT GTG TGG ATC CCA GG-3’
	Reverse:	5′-TAG GGG TTG AAG GAA AGC GG-3’
HPV11 E6	Forward:	5′-TTA GGG TAA CAA GTC TTC CAT GC-3’
(3D-PCR)	Reverse:	5′-ATG GAA AGT AAA GAT GCC TCC A-3’
HPV11 E6	Forward:	5′-TAC CTG TGT CAC AAG CCG TT-3’
	Reverse:	5′-CAG CAG TGT AAG CAA CGA CC-3’
β-actin	Forward:	5′-GGG CAC GAA GGC TCA TCA TT-3’
	Reverse:	5′-AGC GAG CAT CCC CCA AAG TT-3’

### qRT-PCR

HPV11.HaCaT (P9) and A3A-HPV11.HaCaT cells were seeded in six-well plates (1 × 10^6^ cells/well) and incubated for 24 h at 37 °C, 5% CO_2_. Total cellular RNA was extracted and cDNA was synthesized as the above method shown.To quantify level of HPV11 E6 mRNA, qRT-PCR was performed using 2× SYBR Green PCR Master Mix (ABI, USA) and specific primers of HPV11 E6 (Sangon Bio., Shanghai, China), as previously described [25]. The primers for qRT-PCR is described in Table 1. Each sample was performed in triplicate. Relative levels of mRNA were determined by qRT-PCR using ABI Prism 7300 real time PCR system (ABI, Texas, USA).

After seeded and incubated for 24 h, HPV11.HaCaT (P9) were treated for 6 h, 12 h, and 24 h with indicated concentrations of rhIFN-ω. To quantify levels of A3s and E6 mRNA, cDNA was prepared and qRT-PCR was performed using specific primers of A3s (GeneCopoeia Inc., Rockville, MD, USA) and HPV11 E6 (Sangon Bio., Shanghai, China) as the above protocol described. The primers for qRT-PCR are described in Table 1.

To assess the specificity of the PCR amplification, a melting-curve analysis was performed at the end of the reaction. The relative expression levels of targets were calculated by the 2^-ΔΔCT^ method.

### Statistical analysis

All data were expressed as mean ± standard deviation and analyzed using the two-tailed paired Student’s t-test. *P* values less than 0.05 were considered statistically significant.

## Results

### Establishment of A3A-HPV11.HaCaT cells

Several reports have showed that enhanced expression of A3s could result in hypermutation of viral genome including HBV, HIV, HPV. The in vitro HPV11.HaCaT system showed that HPV11 could triggered A3s system (especially A3A), suggesting that A3A might be a key factor in HPV11 mutations [23] Thus, we selected A3A to conduct HPV11 mutation test. The purified lentiviral particles were obtained with its titer (5.02 × 10^8^ IU/mL) after lentiviral packaging and titration. We overexpressed A3A in HPV11.HaCaT cells by using a lentivector-based ORF system and got A3A-HPV11.HaCaT cells after the puromycin selection.

**Fig. 1:**
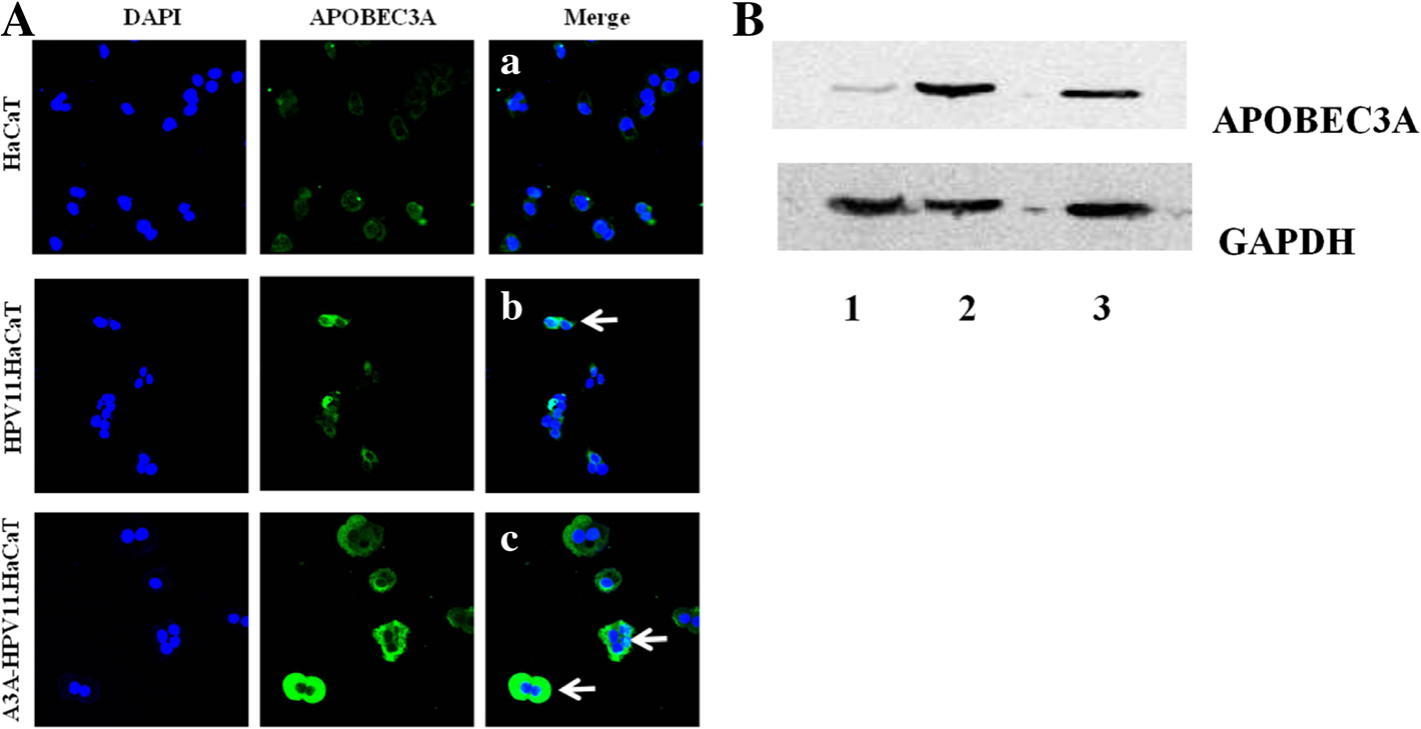
Establishment of APOBEC3A overexpression in HPV11.HaCaT cells (A3A-HPV11.HaCaT). Subcellular localization of APOBEC3A (A3A) in HaCaT, HPV11.HaCaT and A3A-HPV11.HaCaT cells (**a**). Cells were cultured for 24 h prior to fixation and permeabilization. Cells were stained for A3A (green fluorescence) and with DAPI to identify nuclei (blue fluorescence). In the merged panels, white arrows indicate representative cells with increased A3A protein that was allowed to enter in the nuclei. Expression and subcellular distribution of A3A protein in HaCaT (a), HPV11.HaCaT (b) and A3A-HPV11.HaCaT (c) cells. Western blot analysis for A3A expression in HaCaT, HPV11.HaCaT and A3A-HPV11.HaCaT lysates (**b**). HaCaT, HPV11.HaCaT and A3A-HPV11.HaCaT cells were cultured for 24 h. Lane 1: HaCaT cells; lane 2: A3A-HPV11.HaCaT cells; lane 3: HPV11.HaCaT cells

A3A proteins in HaCaT, HPV11.HaCaT and A3A-HPV11.HaCaT cells were detected by immunofluorescence and western blotting. The basal level of A3A protein expression was higher in HPV11.HaCaT cells compared with HaCaT cells (Fig. 1a and b), which was consistent with our previous qRT-PCR results [23]. As observed in the merged images, increased staining of A3A was seen throughout A3A-HPV11.HaCaT cells compared with HPV11.HaCaT cells, especially obvious during telophase [23, 31] (shown as the white arrows in the merged panel, Fig. 1a). Western blots of cell extracts validated the immunofluorescence staining, showing elevated A3A protein in A3A-HPV11.HaCaT cells compared to HPV11.HaCaT cells (Fig. 1b). Both the results showed that A3A-HPV11.HaCaT system was successfully established.

### Growth characteristics of HPV11.HaCaT and A3A-HPV11.HaCaT cells

**Fig. 2:**
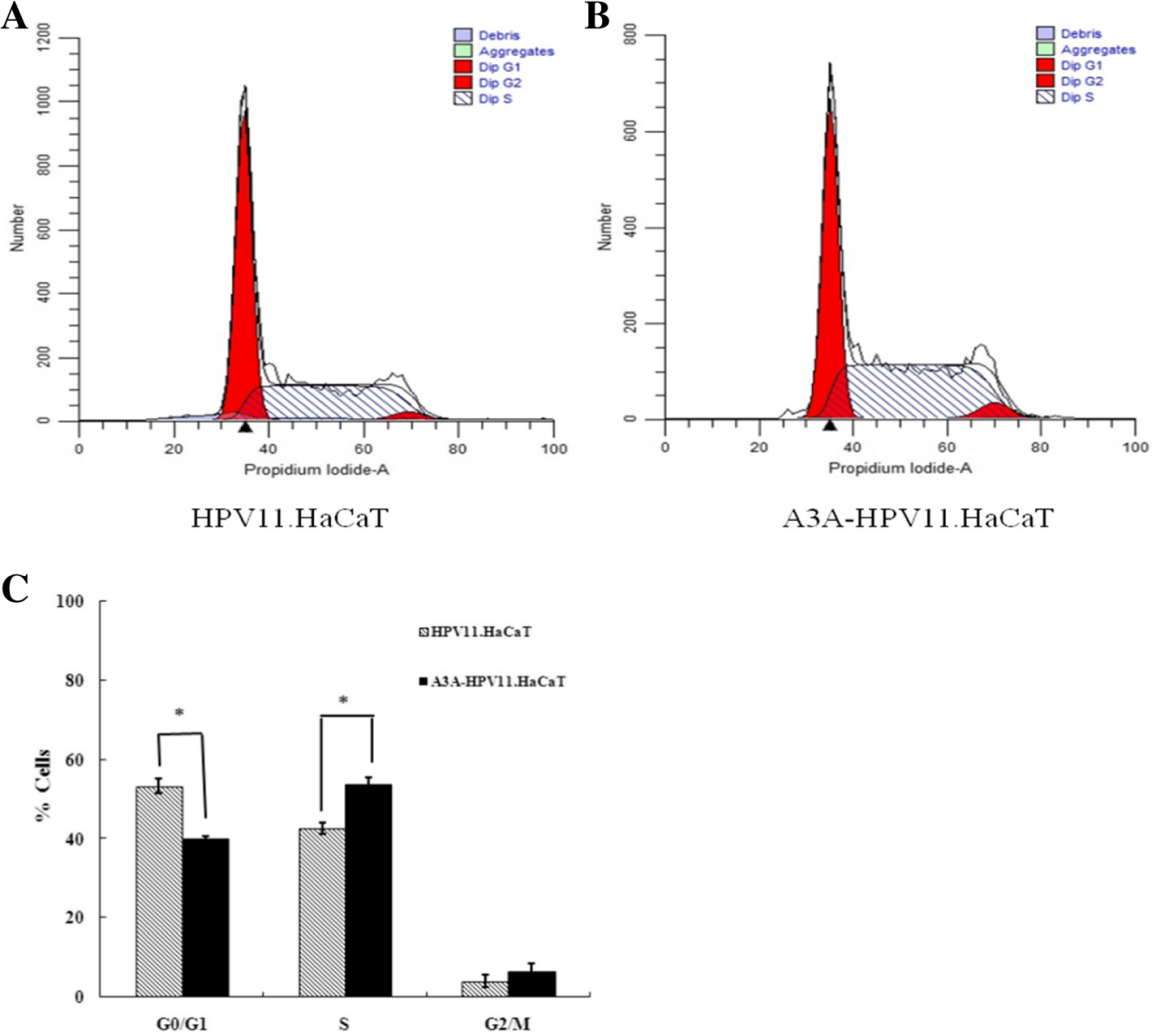
FACS analysis of HPV11.HaCaT and A3A-HPV11.HaCaT cells. Cells were cultured for 24 h prior to digestion and fixation. Cell cycle analysis of HPV11.HaCaT and A3A-HPV11.HaCaT cells was tested by FACS (**a** and **b**). In A3A-HPV11.HaCaT cells, the proportion of cells in G0/G1 phase was obviously decreased, with an obvious increase in S phase (**c**). Data was expressed as means ± SD from three independent experiments. *p < 0.05 vs HPV11.HaCaT using Student *t*-test

Flow cytometry results showed that the proportion of cells in G0/G1 phase was decreased in A3A-HPV11.HaCaT cells, with an increase in S phase compared with HPV11.HaCaT cells (Fig. 2c).

### A3A hypermutated the HPV11 E6 gene

**Fig. 3:**
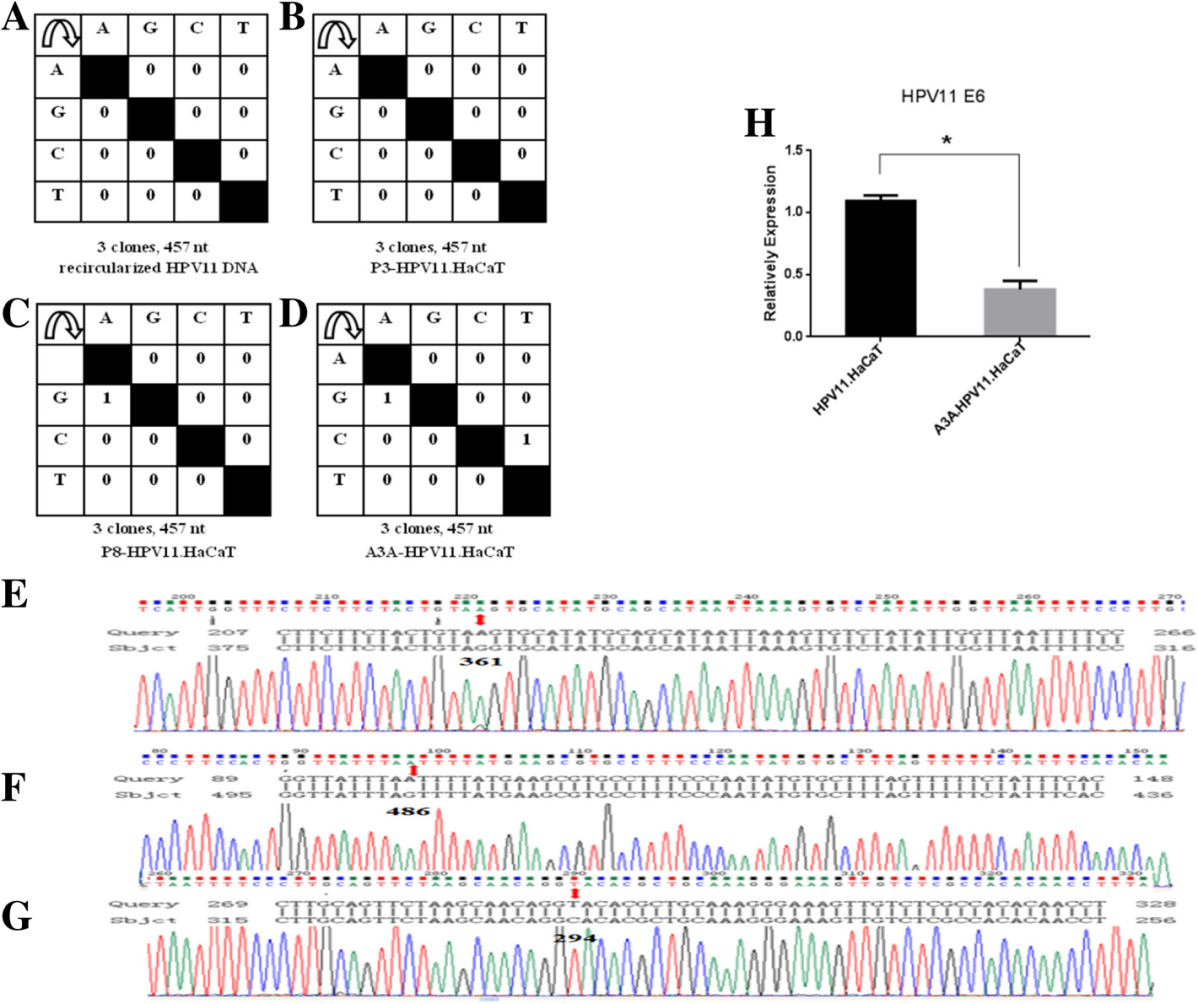
Mutation and the mRNA expression of E6 gene in the recircularized HPV11 DNA, HPV11.HaCaT and A3A-HPV11.HaCaT cells. E6 DNA amplified at a denaturation temperature of 86 °C was excised, cloned, and sequenced. Results are shown as mutation matrices (**a**-**d**) and alignmants (**e**-**g**). The number of C-to-T and G-to-A conversions in each sequence is indicated (**a**-**d**). nt, nucleotides; P3, passage 3; P8, passage 8. Mutated positions of HPV11 E6 gene amplified from P8-HPV11.HaCaT (**e**) and A3A-HPV11.HaCaT (**f** and **g**). qRT-PCR analysis was performed to determine relative E6 mRNA expression in HPV11.HaCaT and A3A-HPV11.HaCaT cells (**h**). Data was expressed as means ± SD from three independent experiments and presented as fold increase in E6 mRNA expression relative to HPV11.HaCaT cells. *p < 0.05 vs HPV11.HaCaT using Student *t*-test

We observed the situation of E6 mutation in A3A-HPV11.HaCaT cells. Firstly, by using 3D-PCR, We amplified HPV11 E6 DNA from the recircularized HPV11 genome used in the establishment of HPV11.HaCaT cells [30] and from HPV11.HaCaT cells (P3). No sign of E6 hypermutation was seen (Fig. 3a and b). Both the results indicated that HPV11 E6 had no mutation in establishment of HPV11.HaCaT cells. Secondly, we detected E6 gene amplified from HPV11.HaCaT cells (P8) and observed a single G > A edited sequence (Fig. 3c and e). The G-A mutant indicated that the HPV11 E6 gene could be edited by long-term HPV11-initiated A3s. Next, we detected E6 mutation positions after A3A-overexpresstion in HPV11.HaCaT cells (P3). We observed that there were some harboring G > A and C > T substitutions among the viral E6 sequences, as shown in Fig. 3d. G > A editing site was present within a TTTAGTTTT sequence motif (edited site underlined, Fig. 3f), while C > T editing site was seen within a CAGGCACAC sequence motif (edited site underlined, Fig. 3g). Above results showed that the HPV11 E6 gene could be edited by enhanced A3A. Finally, qRT-PCR analysis showed that the expression level of HPV11 E6 was obviously decreased in A3A-HPV11.HaCaT cells (Fig. 3h). All the above results indicated that HPV11 E6 might be an A3A target by mutation.

### Up-regulation of A3s expression in HPV11.HaCaT cells by rhIFN-ω stimulation

**Fig. 4:**
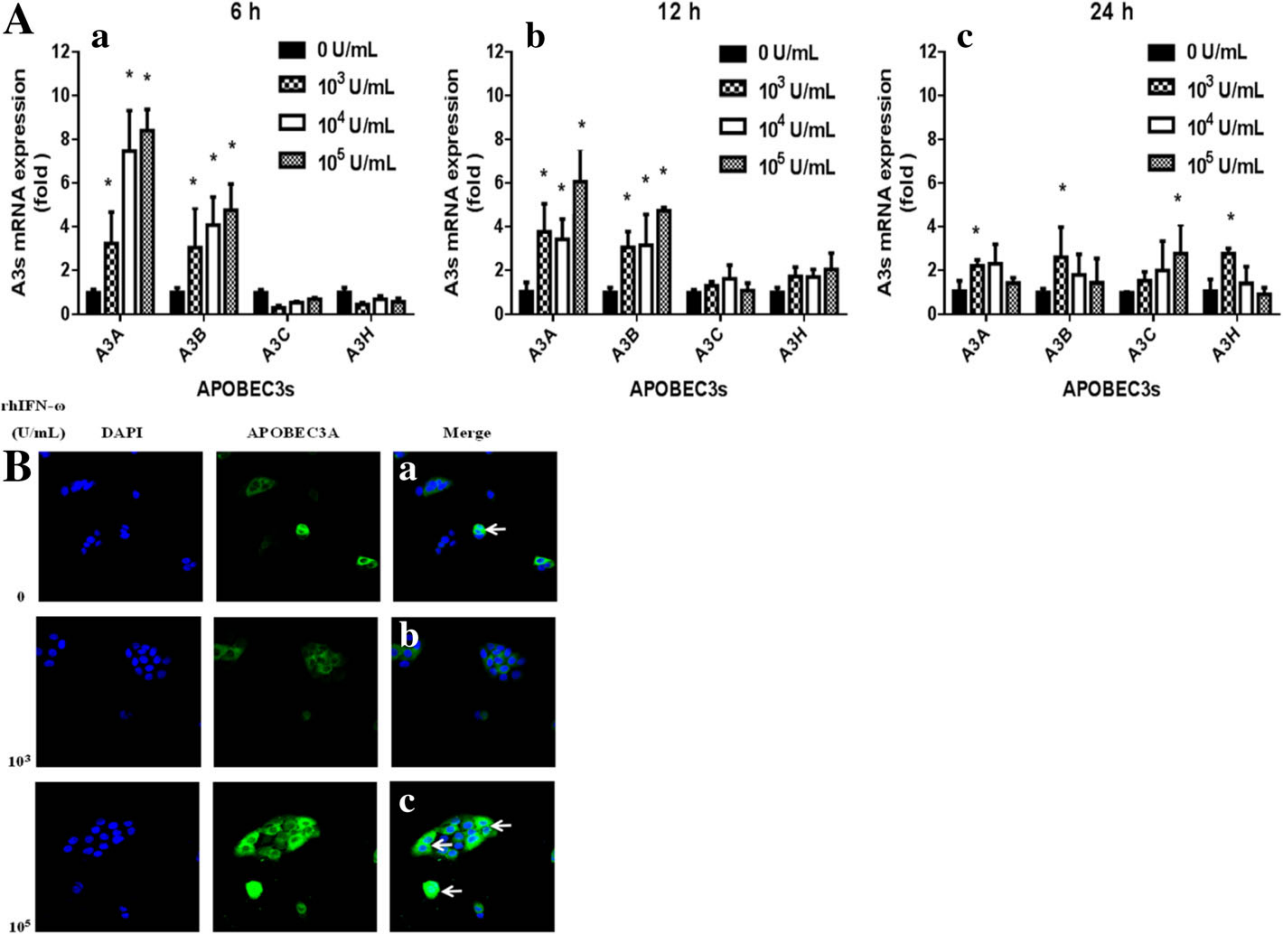
Up-regulation of A3s expression in HPV11.HaCaT cells by rhIFN-ω stimulation. qRT-PCR analysis was performed to determine relative APOBEC3s (A3s) expression levels in HPV11.HaCaT cells (**a**). Cells were treated with rhIFN-ω (10^3^, 10^4^, 10^5^ U/mL) for 6 h, 12 h and 24 h, followed by amplication of APOBEC3s (A3s) mRNA expression. Data was expressed as means ± SD from three independent experiments and presented as fold increase in A3s mRNA expression relative to untreated cells (normal cells). *p < 0.05 vs untreated HPV11.HaCaT using Student *t*-test. A3s mRNA expression after rhIFN-ω treatment for 6 h (a), 12 h (b) and 24 h (c). Expression and subcellular distribution of A3A protein in HPV11.HaCaT cells after treatment with rhIFN-ω by immunofluorescence assay (**b**). HPV11.HaCaT cells were cultured in the presence/absence of rhIFN-ω for 24 h prior to fixation and permeabilization. Cells were stained for APOBEC3A (A3A, green fluorescence) and with DAPI to identify nuclei (blue fluorescence). In the merged panels, white arrows indicated representative cells with increased A3A protein that was allowed to enter in the nuclei (0 U/mL, a; 10^3^ U/ mL, b; 10^5^ U/ mL, c)

qRT-PCR revealed that after stimulation with rhIFN-ω, A3A and A3B mRNA expression (especially A3A) were obviously up-regulated in HPV11.HaCaT cells. Both A3A and A3B mRNA expression arrived maximal levels after treatment for 6 h and their levels were increased with the increasing of rhIFN-ω concentration (Fig. 4A.a). Then their mRNA expression gradually decreased after rhIFN-ω (10^4^ and 10^5^ U/mL) treatmemt for 12 h (Fig. 4A.b) and 24 h (Fig. 4A.c).

Then we detected A3A protein induced by rhIFN-ω in HPV11.HaCaT cells by immunofluorescence assay. The results showed that there existed obvious A3A staining which was mostly localized in the cytoplasm, while it was obviously seen throughout the cells during telophase [31, 32] (indicated as the white arrow in the merged panels, Fig. 4B.a). With the treatment of higher rhIFN-ω (10^5^ U/mL) for 6 h, A3A staining was significantly seen in both the cytoplasm and the nuclei of HPV11.HaCaT cells (shown as the white arrow in the merged panels, Fig. 4B.c).

### Inhibition of E6 mRNA expression by rhIFN-ω

**Fig. 5:**
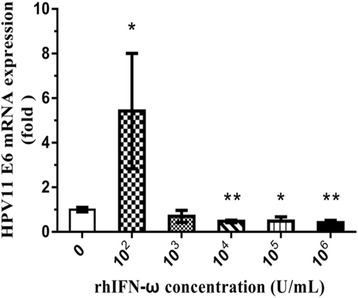
Effects of rhIFN-ω on E6 mRNA expression in HPV11.HaCaT cells. qRT-PCR analysis was performed to determine relative E6 expression levels in HPV11.HaCaT cells. Cells were treated with rhIFN-ω (0, 10^2^, 10^3^, 10^4^, 10^5^, 10^6^ U/mL) for 24 h, followed by amplication of HPV11 E6 mRNA expression. Data was expressed as means ± SD from three independent experiments and presented as fold increase in E6 mRNA expression relative to untreated cells (normal cells). *p < 0.05 or **p < 0.01 vs untreated HPV11.HaCaT using Student *t*-test

Our previous MTT assay results showed that rhIFN-ω at the concentration of 10^2^-10^7^ U/mL didn’t inhibit the proliferation of HPV11.HaCaT cells (data not shown). Based on the results, we chose rhIFN-ω at the concentration of 10^2^-10^6^ U/mL for E6 gene expression tests. RhIFN-ω exhibited increased expression of HPV11 E6 mRNA at 100 U/mL, but significantly inhibited the expression of HPV11 E6 mRNA at 10^4^ U/mL or higher concentration (Fig. 5).

## Discussion

The A3s system is a family of cellular cytidine deaminases that act on single-stranded DNA (ssDNA) or RNA substrates, providing intrinsic immunity to the host against viral infection. The main function of A3s is to inhibit the replication of different viruses through hypermutation of viral genome, such as HIV, HBV and HPV. To date, most studies of A3-mediated hypermutation in HPV have focused on HR genotypes because of their capabilities to cause carcinogenesis. The E6/E7 genetic variations of HPV11 were detected in HPV11 DNA isolated from HPV11-related epithelial lesions [22], but the details involved in these variants still remain unknown. Our previous results indicated that A3s immune system (especially A3A) is triggered by HPV11 [23]. A3A was a powerful restriction factor resulting in inhibition of infection by HPV16 and efficient knockdown of this protein has significantly increased viral infectivity [31]. A3A protein is mostly localized in the cytoplasm, but becomes cell-wide during telophase [32]. Thus we hypothesized that the HPV11 DNA might be vulnerable to editing by some of the A3s, raising a concern about whether enhanced A3A could result in HPV11 E6 hypermutation and could inhibit the expression of E6 gene.

In this study, both the immunofluorescence and western blotting results indicated that A3A-HPV11.HaCaT system was successfully established and might be used for further research. Here we also observed that overexpressed A3A repressed the mRNA expression of HPV11 E6, which implied that enhanced A3A could inhibit the expression of HPV11 early genes.

HPV6 or HPV11 infection resulted in cell cycle disruption which is similar to HR-HPVs infection, although there existed some differences in the expression of P53 and cyclin D1 [33]. HPV18 established its initial infection during S phase, reached its maximum level during G2 phase, and then followed the replication pattern of cellular DNA during S phase in the stable maintenance phase [34]. We previously observed that the proportion of cells in S-phase and G2/M-phase was increased in HPV11.HaCaT cells compared with that in HaCaT cells, with the G1 phase fractions decreased [25]. Here we also observed that enhanced A3A induced S-phase arrest in A3A-HPV11.HaCaT cells. All the above findings indicated that transiently exposed viral ssDNA during replication might have more possibilities to be edited by enhanced A3A activities caused by HPV11 when HPV11 manipulated cell cycle of the host cells to established its replication.

Next, we investigated whether enhanced A3A could hypermutate HPV11 E6 gene. As described above, a single G > A edited sequence was detected in HPV11.HaCaT (P8), while no hypermutant was observed in P3, indicating the presence of the E6 mutant in basal-like HaCaT [30] under the condition of long-term carrying HPV-11 episomes. Thus we speculated that endogenous A3s proteins (especially A3A) triggered by HPV11 might be responsible for this mutation. Then we detected mutation of E6 gene in A3A-HPV11.HaCaT cells. Two C-T/G-A mutants were observed after amplification by 3D-PCR. The above results indicated that HPV11 E6 could be edited by enhanced A3A. The observation that enhanced A3A leaded to a substantial reduction in E6 expression of HPV11 suggested that activated A3A during HPV11 replication is likely to serve a protective function in HPV11.HaCaT cells. Moreover, because the advantage of uracil DNA glycosylase over the activities of cytidine deaminase might cover up the E6 hypermutation [16], further investigations are required to confirm the involvement of A3A in HPV11 E6 mutation by inhibiting uracil DNA glycosylase and transfection of small interfering RNAs against A3A.

Finally we investigated whether A3s family was induced by rhIFN-ω and explored the effects of the agent on E6 gene expression. IFNs are potent mediators involved in cell-to-cell signaling and trigger various pathways to block intracellular replication of viruses and to impede the infection of surrounding cells [35, 36]. IFN-ω, like other IFNs, is a cytokine produced by host cells in defense against viruses and has non-specific antiviral activity [26, 35]. In vitro studies indicated a strong activation of IFN signaling by IFN-ω, but IFN-κ exhibited weaker activation [36]. In this study, rhIFN-ω treatment of HPV11.HaCaT cells could lead to upregulation of A3A mRNA expression and obvious increasement of A3A staining throughout the cells. This agent could also significantly inhibit mRNA expression of HPV11 E6. Based on these results, we speculated that rhIFN-ω might inhibit viral gene expression by enhanced A3A.

All the above results suggested that enhanced A3A might be one of the mechanisms of immunomodulation function and antiviral activities of rhIFN-ω. However we need to further confirm whether there exists mutation of HPV11 episomal DNA in HPV11.HaCaT cells after rhIFN-ω treatment, further studies are required to elucidate the function of A3s in HPV11-related epithelial lesions as well as in anti-HPV11 effects of rhIFN-ω.

## Conclusions

This is the first demonstration that rhIFN-ω upregulated A3A expression and inhibited HPV11 E6 gene. Enhanced A3A repressed HPV11 E6 expression through E6 gene hypermutation, and rhIFN-ω might be an effective agent against HPV11 infection by up-regulation of A3A.

## Abbreviations

3D-PCR: differential DNA denaturation polymerase chain reaction
A3A: APOBEC3A
A3A-HPV11.HaCaT: A3A-overexpressed HPV11.HaCaT
APOBEC3 (A3s): Apolipoprotein B mRNA-editing enzyme catalytic polypeptide-like 3 proteins
CA: condylomata acuminate
Early gene 2/6/7: E2/6/7
HPV11.HaCaT cells: HaCaT keratinocytes containing the genome of HPV 11
HPVs: human papillomaviruses
HR: high-risk
IFNs: Interferons
LR: low-risk
rhIFN-α: Recombinant human interferon-α
rhIFN-ω: Recombinant human interferon-ω
SsDNA: Single-stranded DNA
